# Supplementary material for: Are carbon emissions trading and green financial instruments synergistic? -Comprehensive quantitative research based on content analysis
Source: PLoS One. 2024 Mar 7;19(3):e0298601. doi: 10.1371/journal.pone.0298601 (PMC10919601; doi:10.1371/journal.pone.0298601)
Supplement: S1 Data — (ZIP) [file pone.0298601.s001.zip › Data/地级市Z_F工作报告.zip!/║┌┴·╜¡/╥┴┤║╩╨/└┤╫╘╣1⁄2╓┌║┼í╛┬φ┐╦╩2╛▌═°í┐_2013.docx]

各位代表：

现在，我代表市政府向大会作工作报告，请予审议，并请市政协委员和列席会议的同志提出意见。

一、2012年工作回顾

2012年是新一届政府履职施政的第一年。面对期望与重托，市政府团结带领全市人民，深入贯彻落实市第十一次党代会精神，解放思想，务实进取，攻坚克难，圆满完成了市十三届人大一次会议确定的各项目标任务，全市经济社会呈现增速加快、总量扩大、结构优化、持续向好的发展态势。

——经济实现较快增长。初步预计，全年地区生产总值实现258亿元，同比增长12.1%，增幅较上年提高4.1个百分点。产业结构优化升级。森林食品北药业实现增加值33亿元，跃升为第一大引擎产业。旅游业加速向四季均衡、整体提升方向迈进，全年接待游客543万人次，旅游收入43.6亿元，分别增长12.7%和23.5%。规模以上工业实现增加值44亿元，增长13.6%。内需外需拉动有力。固定资产投资完成221亿元，增长37%，增幅居全省前列。社会消费品零售额实现76.4亿元，增长15.5%。进出口总额完成4.03亿美元，增长15%。收入增幅高于GDP。全口径财政收入实现30亿元，增长22.3%。城镇居民人均可支配收入达到1.4万元，农民人均纯收入突破1万元，分别增长20%和16%。

——生态功能区建设不断深化。认真落实天保二期工程的各项措施，持续推进“严管林”，不断加大森防力度，查处各类资源林政案件102起，依法收回林地120公顷，全年未发生重特大森林火灾。生态移民和自然保护区建设加速推进，撤并林场（所）13个，友好湿地晋升国家级自然保护区。完成污染减排项目16个，万元GDP综合能耗同比下降3.5%。科学调整森林经营方式，更新造林63万亩，森林抚育316万亩，苗圃育苗能力突破2亿株。重新区划落实森林资源管护承包面积316万公顷，营造和改培红松果林2.4万亩、平榛5.8万亩，种植五味子、水飞蓟等地道药材15.7万亩，以蓝莓为主的小浆果种植面积已达3.4万亩，加快了由保护生态向多元化开发利用生态的转变。

——产业项目建设力度持续加大。从年初到岁尾我们紧抓项目不放，围绕加速构建“3+X”产业体系，谋划产业项目170个。全年三次集中开工、五次督导推进，开工建设500万元以上产业项目91个。列入省重点推进的19个产业项目完成投资45亿元，为年度计划的105%，液化空气、双蓝食品等项目相继竣工投产。市县（区）重点推进产业项目72个，比上年增加35个，投资增幅达50.4%。园区建设取得突破，八大产业园区完成基础设施投资7.5亿元，铁力园区入驻项目12个，当年完成投资12亿元。市工业区获批全省唯一生态工业示范基地。

——城市形象和功能较大提升。全年投入城市建设资金77亿元，同比增长51%。棚户区改造大力度推进，圆满完成了省下达的任务目标并通过省政府的核查验收，金山屯、红星、翠峦等地基本完成区局址改造任务。嘉荫、汤旺河、朗乡等旅游名镇影响力不断扩大，梅花山、鹿鸣等特色小镇相继建成。城市改造全面展开，全市新建改建大型主题公园、广场11个。中心城“三个点”、“四个一”等标志性景观加速建设，改造临街建筑外立面39栋，新建改造市政道路9条。全市园林绿化投资1.6亿元，同比增加1亿元，新增各类绿地260公顷。“三供两治”建设力度加大，全市新增供热管网89公里、面积414万平方米，供热普及率达到70%，华能热电开工建设；实施供水项目27个，自来水普及率提高5个百分点；新建续建垃圾处理厂11座，污水处理率达到62%。交通网络不断完善，前嫩公路伊春段建成通车，增开了至广州、天津等航线，伊春与外界的联系更加便捷。

——改革开放呈现新态势。林业资本化运营研究取得阶段性成果。光明股改进展顺利。市农商行挂牌营业。投融资体制改革加速推进，城投平台全年融资23.4亿元，增长1.1倍。对外交流合作务实拓展，先后赴香港、天津、广州、深圳等地开展了系列推介和定向招商活动，参加了亚布力中国企业家论坛年会、厦门投洽会、黑龙江绿色食品北京展销周等经贸活动，强化了与中国人民大学、东北农业大学等名校的合作，组织了“春之约”中国企业家林都伊春行、森博会、农博会、比罗比詹伊春日以及全国学生定向越野锦标赛等节事赛事活动，成功引进汇源、佳龙、阿里洋斯等有实力、信誉好的战略投资者，绿色产业谷、城市综合体等一批重大项目签约落地。全年国内引进到位资金210亿元，实际利用外资5676万美元，增长15%。

——人民生活较大改善。全市地方财政用于民生支出41.8亿元，同比增长40%。开工建设保障性住房8万套438万平方米，惠及20余万人，为3万户住房困难家庭发放廉租住房补贴5000余万元。投入扶贫创业资金544万元，建立示范基地50个，提供岗位3291个；城镇新增就业3.9万人，增加公益性岗位2100个。城镇居民基本医疗保险和新农合补助标准提高到240元。大型养老项目加速推进，新青等福利院建成投用。扶贫济困成效显著，被评为全国五星级慈善城市。改扩建大型社区综合服务中心7个，社区建设经验在全省推广。教育投入8.6亿元，同比增长56%。义务教育标准化学校达标率由上年的58.4%提高到66.7%，高于全省平均水平15个百分点；高考二表以上本科入段率达到44.2%，实现“七连升”。普遍加大了对各级各类医院改造建设力度，中心医院、省林业二院“创三甲”获得成功，县级医院医疗条件继续改善。为部分区局和大型宾馆配备了国际最先进的体检设备，我市成为中国首个拥有多功能无创血液分析仪的城市。平安伊春建设扎实推进，信访和安全生产形势总体稳定。经过全市上下共同努力，市政府年初承诺的17件民生实事全部兑现！

——政府行政效能创新提高。深化“六型”政府机关建设，创新实施政府绩效管理十大工程，政府执行力明显提升。大力开展治浮、治庸、治懒、治慢活动，曝光、查处不作为、慢作为、乱作为行为39起，精简行政审批和许可159项。强化政务公开、网民在线交流和行风热线，群众反映的一些实际问题得到较好解决。加强行政监察和审计监督，“三公”经费同比压缩20.3%。认真落实市人大及其常委会的决议和决定，人大代表议案和政协提案办复率达100%。

此外，民族宗教、外事侨务、双拥共建、人口计生、妇女儿童等各项工作都取得了新成绩。

各位代表，政府过去一年的工作之所以取得较好成效，主要得益于市委总揽全局，为我们指明了前进方向；得益于市人大的依法监督和市政协的建言献策，为我们凝神聚力抓发展提供了有力保障；得益于我们抢前抓早，打出了提前量，赢得了发展主动权；得益于我们创新工作方式，通过实施政府绩效管理工程，一个本子抓落实，一套考评督到底，有效提升了行政效能；更得益于各级干部担当尽责，全市上下干事创业氛围的形成。各地、各部门九牛爬坡个个用力，“5+2”、“白加黑”地大干快上，涌现出了许多创业、创新、创优的典型，有力推动了全市转型跨越发展。在此，我代表市政府，向全市人民，向市人大代表、政协委员，向离退休老领导、老同志，向各民主党派、人民团体和社会各界人士，向驻伊部队和中省直部门，向所有关心和支持伊春发展建设的朋友们，表示衷心的感谢和诚挚的敬意！

我们深知，过去一年全市经济社会发展中出现的向好变化还只是初步的，许多工作还是在打基础、攒后劲、积能量、拓空间。当前，我市仍处在新型工业化、二度城市化、服务业现代化初期，三大引擎产业成长期，解困型、还账型民生向发展型、建设型民生转变期，现代林区经济发展还很不充分。生态功能区的基础比较薄弱，实现绿色崛起仍有很长的路要走。创新发展能力不强，投融资不足仍然是我们亟待破解的难题。城市基础设施不完善，整体配套服务功能还不适应国际化森林生态旅游城市建设的需要。对外开放层次不高，社会建设与管理创新任务繁重。就业门路窄，社会保障压力大。经济发展环境还不够宽松，政府自身建设仍有薄弱环节。我们将认真负责地对待这些问题，集中力量继续攻坚！

二、2013年主要工作安排

今年政府工作的总要求是：深入贯彻落实党的十八大和中央及省市委经济工作会议精神，以科学发展观为指导，进一步突出生态文明建设战略地位，坚持“稳中求快、快中求好”的主基调，把快增长、扩总量作为核心任务，把转方式、调结构作为工作主线，把增收入、惠民生作为根本目的，统筹推进经济、社会、文化和生态建设，为建设美丽富庶、文明和谐、健康幸福新伊春，全面建成林区小康社会奠定坚实基础。

全市经济社会发展的主要预期目标是：地区生产总值增长12%；固定资产投资增长30%；规模以上工业增加值增长12%；地方财政收入增长10%，公共财政预算收入增长13%；社会消费品零售总额增长15%；进出口总额增长10%；城镇居民人均可支配收入和农民人均纯收入分别增长13%和9.8%；城镇登记失业率控制在4.5%以内；万元GDP综合能耗下降3.2%。

为实现上述目标，突出抓好九项工作：

（一）突出绿色发展，打实筑牢赶超跨越的基础。坚定不移地把发展作为核心和首要，用新型工业化的理念和手段加快构建“3+X”产业体系。一是全产业链做强森林食品北药业。坚持保护树种与发展产业并重，以汇源、天隆为龙头，做长红松产业链条，加快把“红松故乡”建成“红松产业之乡”。以创建“省级小兴安岭蓝莓生态科技产业园”为带动，推进蓝莓种植基地化、规模化、现代化，全年新增种植面积3万亩。支持越橘庄园、忠芝等龙头企业在产品深度研发、产业化经营、文化内涵提升等方面下功夫，重点做优做强高档蓝莓红酒，推动“中国山果酒城”加速建设。开展食用菌产业素质提升行动，采取招商引资和政府先进后退等方式，加大产业投入，抓好制菌现代化、栽培工厂化、加工精深化三个环节，在提升品质和价格上实现突破。全年栽培食用菌6.3亿袋。以打造中国名药为方向，扶持格润、葵花等制药企业，将柏子养心片、刺五加浸膏、脑灵素片等疗效显著、已有较高市场知名度的地产药品作为主打产品，强化高端策划营销，叫响“林都北药”品牌，带动北药种植加工全面发展。加强森林食品药品行业规范和自律，严查严堵小作坊、黑加工点，引导现有中小企业挂靠龙头联牌生产。实行企业生产安全承诺制和责任追究制，确保产业健康持久发展。二是围绕“干”字型布局抓好旅游业“五个提升”。提升产品设计和景区建设水平。推出森林冰雪温泉、汤旺河游船观光、森林高尔夫以及鄂伦春部落体验、金祖文化探秘等旅游新产品，推进传统优势景区晋档升级，做强西岭、梅花山、百悦庄、乳影岛、桃源湖、蓝莓酒堡、带岭影视城等一批新景区。提升要素质量。加快住宿设施建设，完善旅游交通网络，加大旅游商品研发力度，打造林都特色菜系，辟建购物一条街、餐饮酒吧一条街，延长旅游链条和游客停留时间，扩大旅游综合拉动效益。提升旅游宣传促销针对性。依托航线大力开发北京、上海、天津、广州、大连、大庆等重点地区；依托哈市客运枢纽，承接全国各地旅游市场；依托高速公路网络，拓展牡丹江、佳木斯、五大连池等省内客源地。提升人才队伍素质。强化实用型旅游人才队伍建设，突出抓好导游员、讲解员、护漂员、滑雪教练员、餐饮服务员的综合素质培训。提升市场监管能力。建立分工负责的旅游市场监管工作机制,规范旅游市场行为。全年接待游客达到580万人次，旅游收入突破50亿元。三是科学发展矿产开发及冶金建材业。坚持保护性开发，加速建设鹿鸣钼矿、翠宏山铁矿和桃山玉矿。深挖地下资源潜力，做好深部、边部找矿，争取开发新矿山。推进西钢和北方水泥产能扩张，全年钢材、水泥产能分别达到500万吨和300万吨。同时，根据产业周期性规律，保护提升家具、人造板、小木制品、纺织等产业。四是大上快建产业项目。全年安排重大产业项目33个，总投资178亿元，年度投资75亿元。省重点推进产业项目19个，年度投资59.2亿元。在强力推进续建项目的同时，重点抓好汇源、中远、晟泰纸业等16个超亿元项目建设，必保达到既定投资进度。五是加快推进园区建设。打破行政区划壁垒，推行“异地共建”、“飞地托管”、“税收互分”的园区发展模式，将新引进的项目科学摆放到八大园区。铁力园区入园项目总量力争超过去年，并争取晋升为省级高新技术产业开发区；翠峦园区至少新落地重大产业项目5个，西林园区争取晋升为省级园区。六是做强工业。在抓好现有规上工业基础上，加快推进新能源轿车、三角翼动力飞机、真空玻璃、电子显示屏等新兴工业发展，年内新增规上企业30户。重视发展地方工业，扶植伊春酱菜、翠峦小烧、乌马河干豆腐等具有浓郁乡土特色的地方名品做强做大。强化工业运行的监控与服务，提高宏观经济增长的稳定性、持续性。

（二）突出生态文明，累积式推进小兴安岭主体生态功能区建设。以建设中国最好的生态功能区为目标，兼顾当前和长远，从“管、抚、退、造、移、治、防”七个方面系统化推进，加速实现绿色崛起。坚持不懈抓严管。不折不扣落实严管资源各项措施，强化野生动植物资源保护，对涉林涉木等违法违纪行为严惩不贷。完善以家庭为基础的管护承包经营责任制，放宽承包期限和面积，通过竞拍、联户经营等方式，逐步建立以大户能人和公司为依托的管护、抚育、造林、防火、林下经营一体化模式，加速实现森林管护全覆盖、责权利三统一。精细化抚育森林。割断抚育与出材的利益链条，建立新型抚育机制。坚持育林为主，科学控制抚育强度与间隔期，促进现有次生林生长。坚持上层抚育与林下更新有机结合，加大针阔叶大苗人工补植力度，促进树种结构优化。坚持生态、经济效益并重，促进林分结构优化，加快第二代阔叶红松林的建成。全年森林抚育314万亩。加速退耕还林还湿。贯彻落实《黑龙江省国有重点林区条例》，依法将农林交错地区行政管理权与资源管理权统一起来，重点抓好北部地区退耕还林，逐步收回被蚕食的林地，守住林地红线。出台湿地保护办法，突出抓好中心城区连片湿地和其它城镇周边湿地的保护。实施造林攻坚战。对林业施业区内的“挂花地”、林缘空地、线路走廊和矿山破坏地采取无偿出让林地经营权等方式，鼓励企业和职工群众分类植树补林。抓好铁力、嘉荫农田林网建设和村屯绿化工作。稳妥推进生态移民。兼顾下山安居与上山就业“两个需要”，积极争取国家移民扶持政策，稳妥推进移民工作，全年撤并林场所13个。统筹治理江河水系。强化黑龙江、汤旺河、伊春河、呼兰河源头保护和沿江沿河生态防护带建设，整体提升对大江大河的水源补给能力。加快实施污水和垃圾处理“打捆”工程，力争用两年时间彻底改善各区局排污问题，促进流域水质整体改善。全年修建江河堤防16公里，治理水土流失2000公顷。同时，着力抓好冶金、建材、能源等行业节能减排，提高企业稳定达标排放率。强化森林生态系统防控。持续加大森防投入，加强设施、队伍、装备、水源地、道路和阻隔带建设。抓好以“天眼工程”为重点的科技防控体系建设，提升功能，扩大在防伐、防垦和有害生物防治等方面的应用范围，保障林地、森林、生态安全。

（三）突出功能品位提升，全面塑造绿色林都新形象。坚持“生态为魂、欧典为型、旅游为绳、宜居为本”的理念，启动实施总投资226亿元的城市建设“十大工程”。保障性安居工程。抓住国家和省加快城镇化建设的机遇，打好棚户区改造攻坚战，突出抓征收拆迁，必保省下达的保障性住房和各区局结转的棚改任务全面完成，实现基本消灭区局址棚户区的目标。同时，根据拆迁需要和市场需求，通过人口集聚和置换，盘活存量房源，推动保障性住房向高层级发展。市政道路建设工程。改造升级青山大街、黎明路等18条老城区道路，建设回龙湾大街、透龙山大街等主干道，推动“三横五纵”路网整体升级。城市绿化工程。动员企事业单位、社区、学校以及社会组织，大力开展垂直、立体和平面绿化。对旅游公路两侧的行道树和灌木丛进行综合治理，间树透景，因地制宜地打造“彩林带”、“彩叶谷”。中心城区对林都大街、新兴大街、青山大街等地段绿化带提档升级，对南北山裸露山体进行修复，增加阔叶、彩叶比重。全市新增绿化面积185公顷。公园广场建设工程。改造提升水上公园、兴安公园、北山公园、伊春河两岸等景观，续建新建中俄友谊公园、丁香主题公园、体育公园等十大公园广场。伊翠铁路拆除工程。做好铁路拆除和易地改线前期工作，适时启动。城市美化亮化工程。对部分山体、广场、楼体等进行亮化，在重要景观区、商业区建设夜景街。对通河路、和平大街等主街路进行综合整饰。“三供两治”工程。加快建设华能热电、新区及翠峦变电站、西山水库水厂、乌马河至新区和林都大街至友好供水管线、中心城区第一净水厂和第二水源地等项目。启动乌马河、翠峦、友好垃圾中转站建设项目，提升垃圾分类处理能力。新区城市综合体工程。抓好规划设计、土地征收、防火飞机场迁移等前期工作，引进战略投资者，分区分项建设。交通运输工程。新建中心区绕城环线及市物流园，加快建设铁金、浩南公路，做好伊嘉高速公路前期工作。旅游基础服务设施建设工程。抓好宏盛、振华、嘉龙等在建星级酒店开复工，年底前全部封顶。推进游客集散中心、景区停车场、自驾游营地、度假村、快捷旅馆建设，完善配套服务，提升接待能力。同时，启动“创文攻坚年”活动，坚持示范创建、环境改造和精细化管理并举，对照标准要求，狠抓薄弱环节，力保明年创建成功。

（四）突出城乡统筹，推动林农一体化发展。以强农、惠农、富农为核心，力促城乡、林农共同繁荣。一是增强农业综合生产能力。大力发展现代农业，优化种植结构，提升产能、品质和效益。抓好“菜篮子”，扩大棚室蔬菜标准化生产规模，提高四季均衡供应能力。全年有机、绿色、无公害食品种植面积达到270万亩。加强灌区、病险水库加固等农田水利设施建设，新增改善灌溉面积2万亩、节水灌溉1万亩。在充分保障土地承包经营权的前提下，引导农民和林业务农职工集约化、专业化经营土地，大力扶持联户经营、专业大户和家庭农场；推广“桃山农园”经验，构建新型市民与农民互信关系，通过提高组织化程度实现农产品与市场的有效对接，力促农民增收。全年新增各类实体型合作社50个。充分发挥雪山来客、翔宇、集佳、鑫宇等龙头企业作用，大力发展生态畜牧业。年内生猪、禽类饲养量分别达到90万头、2700万只，野猪、鹿饲养量分别达到6万头、2.5万头。二是提升林下经济。把发展绿色经济带及绿色园区作为主攻方向和重点，深度挖掘土地、林地的增效潜能，转变种养方式，优化产业布局，全力推进蓝莓、水稻、药材等“七大产业带”和汇源绿色产业谷等“一谷七园”项目建设，力争三年大见成效，实现森林食品北药业的“第二跳”。三是加快推进城镇化建设。进一步推动资源要素向农村配置，支持具备条件的中心村、中心林场所率先实现城镇化，转移人口市民化。抓好规划馆、博物馆、烧柴改革生物质能源试点、标志性建筑、特色小镇“五个一”工程建设。启动整乡整场城镇化建设试点工作，铁力市、嘉荫县各安排1-2个乡（镇），较大区局各安排1个林场（所）推进。加大旧村改造力度，突出抓好公路沿线村屯、场所风貌整治，全面改善住房、给排水、巷道、垃圾处理等基础设施，建设一批具有伊春特色的美丽乡村、美丽林场。

（五）突出创新驱动，激发全社会的创造活力。把创新摆在发展全局的核心位置来抓。一是推进科技创新。强化行业标准制定和知识产权保护，围绕红蓝黑和北药四条产业链中的关键技术开展自主创新，争取在全国率先出台蓝莓、松仁、平榛等种植加工的行业标准和准入条件。围绕八大园区建设布局，同步推进科技创新要素集聚，促进创新成果涌现和扩散。创新与外部智力合作模式，深入开展“百名专家服务行”等活动；推进东北农业大学伊春小浆果及食用菌等基地建设，谋划建设小兴安岭院士创新基地。强化企业技术创新主体地位，打造100家科技型中小企业。重视科学普及，提高全民科技素质。二是推进投融资体制创新。大力推进诚信伊春建设，引导金融机构围绕生态建设、“3+X”产业体系、城市基础设施项目创新金融产品，切实增强金融服务功能。支持农商行做大做强。引进外埠银行和小额担保公司，活化金融市场。推进城投、林业资本化、旅游开发三大平台建设，增强自我融资能力。三是推进人才机制创新。敞开人才入口，除特殊岗位外，一律公开招聘。对教育、医疗、科技、文化、体育等行业，适当调整编制使用权、支配权，按需设岗、以岗定酬。继续强化市校合作，集聚高端人才，培养实用型、技能型人才。设立人才发展专项基金，对有突出贡献的给予重奖。四是深化重点领域改革。完善国有林权制度改革试点，启动活立木产权交易市场。探索林业资本化运营模式，加速推进林业综合改革。以解决森工企业事业化、空心化为着力点，支持林业局建立实体公司，重铸森工企业新辉煌。

（六）突出借力发展，推进务实开放上层级上水平。借力发展是伊春的现实选择，必须热度再升温、力度再加大。一是开展务实招商。以打造“四城一都”为形象化载体，举全市之力招大商、引大企业，推进林区资源与外部市场、资本、人才的深度对接。紧逼加压，落实指标，按市级领导分管战线组团招商。完善招商引资优惠政策，科学调整考评办法，对有突出贡献的给予重奖。全年招商引资200亿元。二是强化以对俄为重点的务实合作。加快建设嘉荫口岸浮箱固冰通道、公路延伸等项目，全力推动口岸复关，打通对俄合作大通道。整合境内外资产、资金、人力等生产要素，组建对俄木材采伐加工联合体，年内境外采伐木材和锯材加工分别达到100万立方米，力争在境外再造一个新林区。依托嘉荫跨境（木业）经济合作园区，打造木材精深加工、农产品、轻工等跨境产业链，促进境内外园区互动发展。加强与阿穆尔州、犹太州在经贸文化旅游等领域的交流合作，提升伊春在俄远东地区开发开放中的影响力。同时，积极扩大与港澳台、东南亚、北美、北欧等国家和地区的交流合作。三是积极对上务实争取。抓住生态文明地位提升机遇，深入研究国家政策导向, 加大衔接沟通力度，重点在国家林业综合改革试验区、森林碳汇交易试点、新能源示范城以及教育、医疗、社保等民生领域的政策争取上取得新突破。

（七）突出财源培育，提高公共财政保障能力。抓住关键环节，构建新型财政体系。一是培育涵养地方主体财源。落实落靠招商引资有关财政税收和非税收入等优惠政策，对本土企业家投资新上产业项目一视同仁，全力支持财源大项目建设。积极做好项目申报工作，争取更多的项目纳入省级财政重点扶持范围。加大投融资力度，完善基础设施，为招商引资提供优良发展环境。发挥财政担保公司和小额贷款公司职能作用，帮助企业解决融资难、贷款难问题。落实增值税、营业税、小型微利企业所得税等优惠政策，善待和支持中小微企业发展，催生规模以上企业。推进政企合一区局财务财政“换位”，做大做强区域财源。二是强化财税征管。在落实好结构性减税政策的基础上，完善部门联动机制，依法加强收入征管，堵塞税收漏洞，确保应征税款及时足额入库。严格执行一站式集中缴费制度，确保非税收入应收尽收。切实保护纳税人的合法权益，坚决防止收取过头税、过头费。三是优化支出结构。坚持综合运筹财力抓关键、保重点。强化预算约束，从严控制预算追加，从严控制拆迁等各种专项资金的使用，从严控制行政机关楼堂馆所建设，推行市场化办节办会，降低政府成本。今年政府系统不更新公务用车，“三公”经费同比压缩20%。

（八）突出社会管理创新，力促社会和谐稳定。社会管理是维护社会和谐稳定的源头性、基础性工作，必须紧抓不放。一是全面推进社区管理创新。总结推广美溪、乌马河、西林、伊春区社会管理创新试点经验，调整优化社区规划布局，合理整合共享资源，加快标准化社区建设，推进社区建设管理工作由点向面延伸。二是强化信访源头预防。坚持依法治访，落实落靠信访责任制，完善社会矛盾排查、决策风险评估、社会化大调处机制，及时掌握社会舆情动态，切实解决群众反映的突出问题。三是抓实公共安全管理。全面落实企业主体、部门监管和属地管理责任，深入开展安全生产标准化达标升级和建设项目“三同时”工作，加强风险管理和应急救援能力建设，有效防范和坚决遏制重特大事故发生。突出抓好食品药品质量监管，保障群众饮食用药安全。深化和完善城市治安防控体系，提升“平安伊春”建设水平，力保在全省率先夺得“长安杯”。

（九）突出民生改善，加速推进城乡公共服务均等化。把人民群众对美好生活的新期待作为工作目标，尽心尽力惠民生。一是促进教育优质均衡发展。加快普及学前教育，整顿规范幼儿教育秩序。调整学校布局，年末义务教育阶段标准化学校达到75%。提升普通高中教育水平，力争高考本科二表以上入段率达到45%以上。做优做强市一中等省级示范性高中，提高名校的吸引力和带动力。加大教师进修学院软硬件投入，提高师资培训能力和水平。优化职教资源，支持职业学院“专升本”，支持伊春林校创建国家中等职业教育改革发展示范校，打造特色专业和品牌学校。建立链条式保障机制，用好市公共实训基地。依托电大建立终身教育体系。二是推动更高质量就业。坚持把充分就业作为重要前提，努力营造公平的就业环境。强化培训，提升创业就业能力，引导高校毕业生、农村转移劳动力、城镇困难人员、退役军人等重点群体向旅游、木艺、玉石加工等高报酬行业集中。强化创业扶持政策落实，抓好税费减免、小额贷款发放、基地建设等工作，为创业就业创造条件、搭建平台。三是提升医疗卫生服务水平。深化医药卫生体制改革，巩固完善基本药物制度，切实减轻群众医药负担。充分发挥三甲医院的带动作用，推进各级各类医院提档升级，争创特色品牌医院。四是健全社会保障体系。扩大社会保险覆盖面，建立健全社会保障增长机制，与全省同步提高社会保险待遇和城乡低保水平。顺应我市老龄化趋势，加快推进以居家养老为基础、社区服务为依托、机构养老为补充的养老服务体系建设。做好优扶安置工作，完善社会救助制度，争创“六星级慈善城市”。五是大力发展文体事业。深入推进“6541”工程，加快发展文化事业和文化产业，繁荣小兴安岭文化。重点抓好公共图书馆、群众艺术馆和社区文化场所建设，不断满足城乡居民精神文化需求。以举办第十三届省运会为契机，加快体育场馆及全民健身设施建设，培育冰壶、滑雪、漂流、越野等品牌赛事，打造“运动之城”。

今年，市财政用于民生方面的支出同比增长10%以上，重点办好20件民生实事：

1、开工建设保障性住房3.2万套180万平方米，改造泥草房6990户，建设公租房1000套。

2、落实国家和省出台的调资政策，提高职工工资水平。

3、逐步解决厂办大集体职工的养老统筹问题。

4、提高住房公积金匹配比例。

5、对林城、红升、制材等旧小区进行综合改造，重点解决水电管网等“内设施”老化问题。

6、提升供热供水能力，全市供热、自来水普及率分别提高3个和2个百分点。

7、建设中心城区和金山屯燃气管网。

8、全面完成公办幼儿园建设任务，开园率达到90%以上。

9、免收90%中职学校学生学费，对20%普通高中学生给予助学补助，对1000名考入本科二表以上困难学生给予一次性资助。

10、城市低保月人均标准提高到301元，农村低保标准年人均提高到1718元。

11、为市直机关、事业单位聘用的临时用工人均月增资300元。

12、提高1—6级工伤人员伤残津贴标准，平均每人每月增加500元。

13、救助困难职工4000人，开发公益性岗位2000个以上，免费培训创业人员1500人，发放小额贷款2000万元。

14、将企业长期临时用工和机关事业编制外人员纳入城镇职工基本养老保险。

15、将城镇职工和居民基本医疗保险、企业职工生育保险纳入市级统筹，城镇职工和居民基本医疗保险政策内报销比例力争分别达到80%和70%。

16、投入5100万元，改善县（市）区医疗设备条件。

17、将政企合一区局和“南四局”纳入国家基本公共卫生服务范围，免费提供老年人保健等10类41项公共卫生服务。

18、取消和免征户口本工本费、个体工商户注册登记费、房屋租赁管理费等30项涉及企业和居民的行政事业性收费。

19、新建改造300平方米以上社区10个。

20、增开中心城区公交线路、延长营运时间。

三、加强政府自身建设

全面完成今年各项目标任务，关键在于提高各级政府的公信力和执行力。一要继续实施绩效工程。把绩效管理作为落实市委决策及重点工作的主抓手，总结经验和不足，完善推进机制，在全市政府系统全面铺开。二要勇于担当尽责。政府班子要率先垂范，讲责任、重细节、抓落实。大力推行一把手一线工作制、部门配合联动制，对市委、市政府确定的各项工作，尤其是在打好“五个硬仗”上，要敢啃硬骨头，敢碰关键点，切实解决“最后一米”落实不下去问题。三要坚持依法行政。严格执行重大决策集体研究、专家咨询和听证制度，提高政府依法、科学、民主决策水平。自觉接受人大法律监督、政协民主监督，认真办理人大代表建议和政协提案，充分听取社会各界的意见建议。扎实推进政府信息公开，切实提高政府工作透明度。四要保持风清气正。认真落实党风廉政建设责任制，强化廉政风险防控和审计监督。对照中央“八项规定”，务实转变作风。今年，要把为基层减负、为基层服务作为重点，禁止政府部门巧立名目下基层，大力精简行政审批和许可，从严控制会议数量、规模和时间，各类专项工作一律以电子公文形式部署和反馈，市政府及县区行政主要领导原则上不参加专业性会议，把主要精力用到谋发展、抓招商、上项目、惠民生中去。

各位代表，2013这张新考卷等待着我们用勇气、智慧和实干来作答。我们坚信，有市委的坚强领导，有全市上下的群策群力，有各级政府的担当尽责，锐意进取，本次会议确定的各项任务一定能够圆满完成，伊春与全省、全国同步建成小康社会的目标也一定能够早日实现！
